# Effects of long-term treatment with testosterone on weight and waist size in 411 hypogonadal men with obesity classes I-III: observational data from two registry studies

**DOI:** 10.1038/ijo.2015.139

**Published:** 2015-08-25

**Authors:** F Saad, A Yassin, G Doros, A Haider

**Affiliations:** 1 Global Medical Affairs Andrology, Bayer Pharma, Berlin, Germany; 2 Department of Urology, Gulf Medical University, Ajman, UAE; 3 Institute for Urology and Andrology, Segeberger Kliniken, Norderstedt, Germany; 4 Department of Preventive Medicine, Men's Health Program, Dresden International University, Dresden, Germany; 5 Department for Epidemiology and Statistics, Boston University School of Public Health, Boston, MA, USA; 6 Private Urology Practice, Bremerhaven, Germany

## Abstract

**Background/Objectives:**

Long-term testosterone replacement therapy (TRT) up to 5 years has been shown to produce progressive and sustainable weight loss (WL) in hypogonadal men. This study investigated effects of long-term TRT up to 8 years in hypogonadal men with different obesity classes.

**Subjects/Methods:**

From two independent observational registries we identified a total of 411 obese, hypogonadal men receiving TRT in urological clinics. The effects of TRT on anthropometric as well as metabolic parameters were studied for a maximum duration of 8 years, mean follow-up: 6 years. All men received long-acting injections of testosterone undecanoate in 3-monthly intervals.

**Results:**

In all three classes of obesity, T therapy produced significant WL, decrease in waist circumference (WC) and body mass index (BMI). In patients with class I obesity, mean weight decreased from 102.6±6.4 to 84.1±4.9 kg, change from baseline: −17.4±0.5 kg and −16.8±0.4%. WC in this group of patients decreased from 106.8±7.4 to 95.1±5.3 cm, change from baseline: −10.6±0.3 cm. BMI decreased from 32.69±1.4 to 27.07±1.57, change from baseline: −5.52±0.15 kg m^−2^. In patients with class II obesity, weight decreased from 116.8±6.9 to 91.3±6.3 kg, change from baseline: −25.3±0.5 kg and −21.5±0.4%. WC decreased from 113.5±7.5 to 100.0±5.4 cm, change from baseline: −13.9±0.4 cm. BMI decreased from 37.32±1.45 to 29.49±1.71, change from baseline: −8.15±0.17 kg m^−2^. In patients with class III obesity, weight decreased from 129.0±5.6 to 98.9±4.8 kg, change from baseline: −30.5±0.7 kg and −23.6±0.5%. WC decreased from 118.5±5.6 to 103.8±4.9 cm, change from baseline: −14.3±0.4 cm. BMI decreased from 41.93±1.48 to 32.46±1.59, change from baseline −9.96±0.29 kg m^−2^.

**Conclusions:**

Testosterone therapy appears to be an effective approach to achieve sustained WL in obese hypogonadal men irrespective of severity of obesity. Based on these findings we suggest that T therapy offers safe and effective treatment strategy of obesity in hypogonadal men.

## Introduction

Obesity is a major public health threat that has an enormous economic burden on society, with an estimated economic impact of greater than $2 trillion.^1^ In the USA ~35.5% of adult men and 35.8% of adult women are obese (BMI ⩾30 kg m^−2^).^2, 3^ Obesity increases risks for atherosclerosis, diabetes, metabolic syndrome, nonalcoholic fatty liver disease, heart disease among other comorbidities and reduces life expectancy.^2^ Obesity contributes to pathophysiological conditions such as hemodynamic, arrhythmic and anatomical modifications in the cardiovascular system.

Contrary to the previously held views, obesity is a chronic disease necessitating medical intervention. If left untreated, obesity contributes significantly to a host of adverse effects on the cardiovascular system. Thus, significant weight loss (WL) at any time during adult life may result in cardiovascular benefit.^4^ It is becoming clear that simple behavioral and lifestyle approaches, such as diet and exercise alone, while considered a first step in management of obesity, are inadequate and for the most part unsuccessful for treatment of obesity, especially in the long run. The limited success of diet and lifestyle in the treatment of obesity and cardiovascular disease (CVD) has led to the termination of the Look AHEAD Trial, suggesting that lifestyle changes alone are insufficient and medical intervention is deemed necessary.^5^ New strategies are urgently needed to combat and manage obesity.^2^ Although attempts to manage obesity with lifestyle changes and therapeutic interventions have been made frequently and are successful to some extent, weight regain remains a major problem.^5^ A proactive approach is necessary for the treatment of obesity in order to reduce the onset or complications of other comorbidities such as type 2 diabetes mellitus (T2DM) and CVD.^6^

A wealth of evidence exists demonstrating that WL improves health outcomes and reduces healthcare costs.^7^ WL is associated with reduction in the incidence of hypertension and high triglycerides, concomitant with reduction in cardiovascular mortality.^8^ Although pharmacological approaches in the treatment of obesity are met with mixed success, employment of surgical approaches has taken hold. Bariatric surgery has been shown to be successful in selected patients and has proven useful in the management of excessively obese patients. Gastric by-pass surgery reduced mortality by ~29% and decreased deaths from CVD.^9^ T2DM was significantly reduced by surgical intervention and other approaches of WL.^10, 11, 12^ Arterburn *et al.*^13^ demonstrated reduced rates of mortality and decreased deaths from CVD in patients who underwent bariatric surgery. The benefit of WL on diabetes was demonstrated in the Look AHEAD Trial.^14^ In the Swedish Obese Subjects study, CVD was reduced by WL.^9^ It should be emphasized, however, that bariatric surgery is not available to all obese patients and not without risks.^15^

Current strategies for treatments for obesity include diets, exercise, behavioral lifestyle changes, pharmaco-therapeutic agents and bariatric surgery.^16, 17, 18^ Treatment of obesity with incretin, glucagon-like peptide-1 (GLP-1) receptor agonists, enzyme inhibitors, angiopoietin-like proteins has been investigated. Many of these approaches have yielded modest but not fully sustainable WL. In contrast, bariatric surgery has provided promising results.^19^

Obesity contributes to the decline of testosterone (T) and the prevalence of hypogonadism is greater than 70% in men with excessive obesity.^20^ T therapy in men with T deficiency (TD) reduces fat mass, increases lean body mass with concomitant WL, reduction in waist circumference (WC) and body mass index (BMI).^21, 22, 23, 24, 25, 26, 27, 28, 29, 30, 31, 32, 33, 34, 35, 36, 37, 38, 39, 40, 41, 42, 43^ T therapy in hypogonadal obese men has been suggested as a novel approach for the treatment of obesity.^44, 45^ Long-term T therapy in men with TD reported significant and sustained WL, reduced BMI and WC.^35, 36, 37, 38, 39, 40^ The potential implication of T therapy in management of obesity in men with TD needs to be explored. T therapy produced sustained WL without recidivism.^35, 36, 38^ It is possible that T therapy in obese men with TD may prove useful in treatment of the underlying patho-physiological conditions of obesity. In this report, we summarize our findings on long-term T therapy in men with TD with varying classes of obesity. The data presented suggest significant improvement in WL, reduction in WC and BMI. We propose use of T treatment as a novel therapeutic strategy for managing overweight and obesity in hypogonadal men with TD.

## Patients and methods

### Patients

From two prospective, cumulative registry studies of 622 hypogonadal men, we identified all 411 obese hypogonadal men (66.1% of all patients) with varying classes of obesity (class I (BMI 30–34.9; *n*=214, mean age: 58.61±8.04 years), class II (BMI 35–39.9; *n*=150, mean age: 60.35±5.73 years) and class III (BMI ⩾40 kg m^−2^; *n*=47, mean age: 60.51±5.52 years). All men were treated with testosterone undecanoate injections (TU; Nebido, Bayer Pharma, Berlin, Germany) for up to 8 years. Men were entered into the registry once they had received 1 year of treatment. Therefore, registry participants had been in the registry for different durations of time, and declining numbers do not reflect drop-out rates.

Inclusion criteria were two separate morning measures of total testosterone ⩽12.1 nmol l^−1^ and the presence of hypogonadal symptoms measured by the Aging Males' symptoms scale (AMS).

Exclusion criteria for testosterone administration included previous treatment with androgens, prostate cancer or any suspicion thereof, such as prostate-specific antigen levels >4 ng ml^−1^ or abnormal findings upon digital rectal examination, International Prostate Symptom Score (IPSS) >19 points, breast cancer, a history of congestive heart failure or recent angina, or severe untreated sleep apnea.

### Assessment and follow-up

During this period, we evaluated the effects of long-term T therapy on the following parameters: total plasma T levels, weight, waist circumference (WC), BMI, hemoglobin, hematocrit, fasting glucose levels and hemoglobin A_1c_ (HbA_1c_), systolic blood pressure (SBP) and diastolic blood pressure (DBP), lipid profile (total cholesterol, low-density lipoprotein (LDL) cholesterol, high-density lipoprotein (HDL) cholesterol, triglycerides), C-reactive protein (CRP) and liver transaminases. We also assessed the effects of T therapy on prostate volume, prostate-specific antigen and questionnaires IPSS, AMS and the International Index of Erectile Function, Erectile Function domain (IIEF-EF). Measures were taken between two and four times per year and annual average was calculated. One patient with obesity class I dropped out after 39 months of treatment as a result of moving to a different geographical location.

Ethical guidelines as formulated by the German ‘Ärztekammer' (the German Medical Association) for observational studies in patients receiving standard treatment were followed. After receiving an explanation regarding the nature and the purpose of the study, all subjects consented to be included in the research of their treatment protocol.

The data from these 411 obese, hypogonadal men were included in this analysis. Statistical methodology was described previously.^35^

## Results

### Baseline characteristics

Table 1 provides baseline characteristics of obese patients stratified to various classes of obesity based on BMI. In class I (*n*=214), 33.2% had prediabetes, defined by HbA_1c_ of 5.7 to 6.4%, 32.7% had T2DM and 6.1% had type 1 diabetes mellitus (T1DM). History of myocardial infarction was 2.8% and history of stroke was 0.9%. In class II (*n*=150), 19.3% had prediabetes, 51.3% had T2DM and 5.3% had T1DM. History of myocardial infarction was 11.5% and history of stroke was 3.1%. In class III (*n*=47), 6.3% had prediabetes, 55.3% had T2DM and 4.3% had T1DM. History of myocardial infarction was 23.4% and history of stroke was 2.1%.

### Effects of long-term T therapy on circulating total T levels in men with various classes of obesity

**Figure 1:**
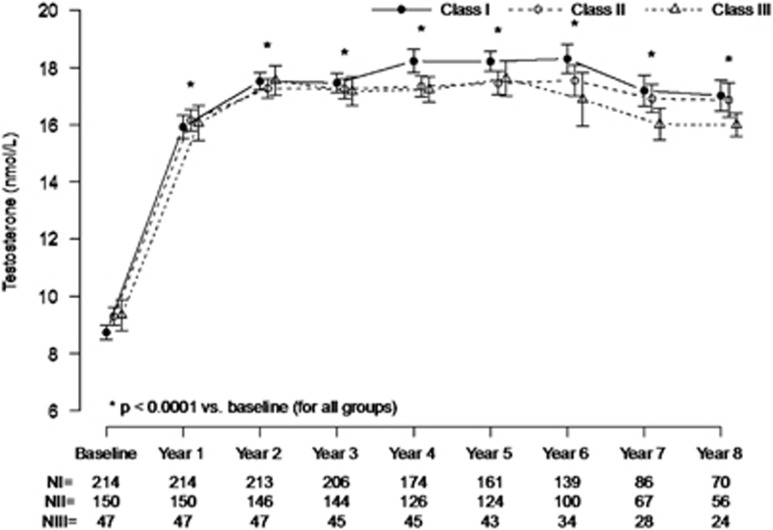
Trough levels of total testosterone (mean±s.e.) in 411 hypogonadal men in obesity classes I, II, and III receiving long-term testosterone treatment.

Figure 1 shows that irrespective of the class of obesity, TU treatment of obese patients restored total T levels within the physiological range during the first year and these levels were maintained in the physiological range over 8 years of follow-up.

### Effects of long-term T therapy on the anthropometric parameters in men with various classes of obesity

**Figure 2:**
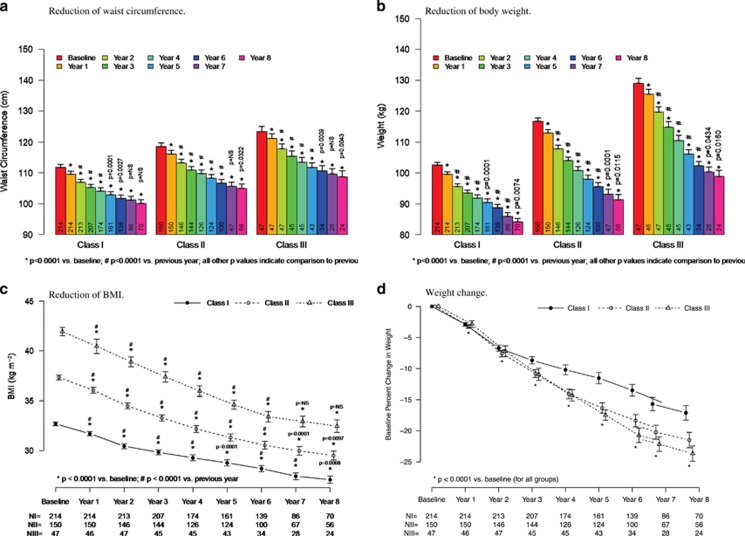
Reductions of waist circumference (**a**), body weight (**b**), BMI (**c**) and weight change (**d**) in 411 hypogonadal men receiving long-term testosterone treatment. All values are shown as mean±s.e.

As shown in Figures 2a–d and Table 2, in all three classes of obesity, T therapy produced significant WL, decrease in WC and BMI. In patients with class I obesity, mean weight decreased from 102.6±6.4 to 84.1±4.9 kg; the changes in weight were statistically significant for all 8 years vs previous year. The change from baseline was −17.4±0.5 kg and the percent change from baseline −16.8±0.4%. WC in this group of patients decreased from 106.8±7.4 to 95.1±5.3 cm. The changes were statistically significant for 6 years vs previous year. Mean change from baseline was −10.6±0.3 cm. BMI decreased from 32.69±1.4 to 27.07±1.57, mean change from baseline −5.52±0.15 kg m^−^^2^.

With regard to WL, in class I, 200 patients (93.5%) lost ⩾5% of their baseline weight, 144 (67.3%) ⩾10%, 85 (39.7%) ⩾15%, 35 (16.4%) ⩾20%, 6 (2.8%) ⩾25% and 3 patients (1.4%) gained weight (Table 3).

In patients with class II obesity, weight decreased from 116.8±6.9 to 91.3±6.3 kg. The changes in weight were statistically significant for all 8 years vs previous year. The change from baseline was −25.3±0.5 kg, percent change from baseline −21.6±0.4%. WC decreased from 113.5±7.5 to 100.0±5.4 cm. The observed changes were statistically significant for the first 6 years vs previous year. The mean change from baseline was −13.9±0.4 cm. BMI decreased from 37.32±1.45 to 29.49±1.71, mean change from baseline −8.15±0.17 kg m^−1^^2^ (Figures 2a–d; Table 2).

Examining WL in patients in class II, 147 patients (98%) lost ⩾5% of their baseline weight, 134 (89.3%) ⩾10%, 108 (72%) ⩾15%, 65 (43.3%) ⩾20%, 19 (12.7%) ⩾25%, 3 men (2%) ⩾30% and no patient gained weight (Table 3).

In patients with class III obesity, weight decreased from 129.0±5.6 to 98.9±4.8 kg. The changes were statistically significant for all 8 years vs previous year (Figures 2a–d; Table 2). The change from baseline was −30.5±0.7 kg and the percent change from baseline −23.6±0.5%. WC decreased from 118.45±5.64 to 103.75±4.86 cm. Changes were statistically significant for the first 6 years vs previous year. The mean change from baseline was −14.3±0.4 cm. BMI decreased from 41.93±1.48 to 32.46±1.59, mean change from baseline −9.96±0.29 kg m^−^^2^ (Figures 2a–d; Table 2).

In class III obesity, all 47 patients lost ⩾5% of their baseline weight, 45 (95.7%) ⩾10%, 42 (89.4%) ⩾15%, 28 (59.6%) ⩾20%, 11 (23.4%) ⩾25% and no patient gained weight (Table 3).

### Effects of long-term T therapy on the metabolic parameters in men with various classes of obesity

Table 2 summarizes the findings of this study with regard to the changes in metabolic parameters and improvement in quality of life in men with varying classes of obesity. In men with class I obesity, long-term T therapy resulted in decreased fasting blood glucose from 5.97±1.65 to 4.95±0.47 mmol l^−1^. The change from baseline was −0.86±0.10 mmol l^−1^. A reduction in HbA_1c_ was recorded from 6.67±1.25 to 5.37±0.4%, and a change from baseline of −1.15±0.06%. Total cholesterol (TC; mmol l^−1^) decreased from 7.01±1.12 to 4.73±0.28, LDL (mmol l^−1^) from 4.14±0.84 to 2.48±0.79, triglycerides (TG; mmol l^−1^) from 2.98±0.68 to 2.04±0.23. HDL (mmol l^−1^) increased from 1.21±0.41 to 1.67±0.37. The TC/HDL ratio declined from 6.45±2.44 to 2.96±0.59. SBP (mm Hg) decreased from 144.3±14.59 to 125.91±8.37, DBP from 85.2±10.38 to 74.18±4.69. CRP (mg dl^−1^) declined from 2.15±2.23 to 0.34±0.34 (*P*<0.0001 for all).

In men with class II obesity, long-term T therapy reduced fasting glucose from 6.24±1.43 to 5.08±0.46 mmol l^−1^. The change from baseline was −2.80±0.10 mmol l^−1^. HbA_1c_ levels were reduced from 7.5±1.32 to 5.78±0.56%. The change from baseline was −1.79±0.08%. Concentrations of TC decreased from 7.60±1.04 to 4.78±0.25, LDL from 4.53±0.72 to 2.89±0.73, TG from 3.32±0.66 to 2.10±0.16 and HDL increased from 1.45±0.50 to 1.99±0.48. The TC/HDL ratio declined from 6.07±2.87 to 2.55±0.62. SBP (mm Hg) decreased from 157.39±15 to 129.15±8.13, DBP from 93.54±12.03 to 74.33±5.44. CRP (mg dl^−1^) declined from 3.23±4.39 to 0.41±0.65 (*P*<0.0001 for all).

Similarly, in men with class III obesity, T therapy produced marked reduction in fasting glucose, which decreased from 6.40±1.31 to 5.18±0.32 mmol l^−1^. The change from baseline was −1.19±0.16 mmol l^−1^. T therapy resulted in reduction in HbA_1c_ from 7.56±1.37 to 5.68±0.44%, and a change from baseline of −1.87±0.13%. Significant reductions occurred in TC from 7.94±1.10 to 4.81±0.23, LDL from 4.95±0.94 to 3.13±0.64 and TG from 3.72± 0.68 to 2.15±0.12. We observed an increase in HDL from 1.60±0.51 to 2.18±0.41. The TC/HDL ratio declined from 5.66±2.88 to 2.29±0.47. SBP (mm Hg) decreased from 161.3±14.25 to 132.7±8.12, DBP from 97.06±10.79 to 75.97±5.43. CRP (mg dl^−1^) declined from 3.73±4.28 to 0.29±0.31 (*P*<0.0001 for all) (Table 2).

### Effects of long-term T therapy on the quality of life parameters in men with various classes of obesity

As shown in Table 2, T therapy resulted in significant improvement in quality of life as assessed by the marked and significant reduction in the AMS (32-point reduction in class I; 33-point reduction in class II and 38-point reduction in class III) (Table 2). Similarly, a significant reduction was recorded in IPSS in all classes of obesity, indicating that T therapy improves lower urinary tract symptoms in obese men and also improves urinary flow. More importantly, a higher score was recorded for the International Index of Erectile Function (IIEF-EF) suggesting that T therapy improves erectile function in obese men, irrespective of the class of obesity (Table 2).

### Effects of long-term T therapy in men with various classes of obesity according to age

We have performed subgroup analyses to assess the effectiveness of T therapy in patients ⩽65 years old (*n*=323) and in patients >65 years old (*n*=88). As shown in Table 4, T therapy appears to be equally effective in WL, reduction in WC and BMI in both subgroups, as in all other parameters, irrespective of age.

### Effects of long-term T therapy on the safety parameters in men with various classes of obesity

T therapy in obese men increased hemoglobin concentrations and hematocrit but the levels remained within physiological ranges (Tables 2 and 4). There were no reported major adverse cardiovascular events. T therapy in men in all three classes of obesity increased prostate volume. However, no case of urinary retention was reported. In fact, lower urinary tract symptoms decreased, as assessed by the IPSS scale (Tables 2 and 4). As expected, serum prostate-specific antigen increased in all men in the three classes of obesity but the increase was not deemed clinically meaningful. Eight patients were diagnosed with low-grade prostate cancer while on T treatment.

## Discussion

In this study, we report that in men with various classes of obesity and TD, long-term T therapy produced significant and sustained WL, together with marked reductions in WC and BMI. Furthermore, we demonstrate that long-term T therapy in men with various classes of obesity reduced blood glucose, HbA_1c_, SBP and DBP, CRP and improved lipid profiles. We also note that long-term T therapy in men with various classes of obesity resulted in significant reduction in alanine and aspartate transaminases suggesting reduced fat content in the liver and improvement in liver function. The marked and significant improvements in various metabolic parameters clearly indicate improvement in metabolic function, as reflected by decrease in inflammatory biomarkers and improved liver function. These findings combined with the improvement in lipid profiles, blood sugar, blood pressure and urogenital function support the reported improvement in quality of life assessed by the AMS questionnaire and the improvement in lower urinary tract symptoms assessed by the IPSS questionnaire. Equally important is the improvement observed in erectile function, assessed by the IIEF (EF) scale. The data from subgroup analyses in patients ⩽65 years old or >65 years old demonstrated that T therapy is equally effective in improving the anthropometric parameters as well as the metabolic functions in both subgroups, irrespective of age, as suggested previously.^46^ Therefore we emphasize that long-term T therapy is effective in younger as well as older patients, contrary to some previous claims. This is an important finding that indicates benefits of T therapy are not limited by age.

The improvements in the cardio-metabolic risk factors are, in part, attributed to improved metabolism, mitochondrial function, increased energy utilization, reduced inflammation, increased motivation and vigor resulting in improved cardio-metabolic function and enhanced physical activity.^45^ The improved motivation, vigor, energy and reduced fatigue associated with long-term T therapy is likely to have contributed, in part, to the observed WL in obese men.^23, 25, 26, 31, 34, 36, 37, 38, 47, 48^ The significance of our findings is that long-term T therapy in hypogonadal men with varying classes of obesity may represent a novel effective and safe intervention strategy in management of obesity in hypogonadal men.^49^

As obesity is a chronic disease, necessitating medical intervention and long-term therapy, it is imperative to develop new approaches to the management of obesity.^2^ Recently, treatment with liraglutide coupled with a diet and exercise resulted in sustained and significant WL. This treatment also reduced cardiovascular risk in obese nondiabetic adults. Treatment with liraglutide produced reductions in SBP and fasting blood glucose, HbA_1c_ and reductions in CRP concentrations.^50^

One of the recent advances in management of obesity is bariatric surgery. This approach has produced substantial and sustained WL and ameliorated several obesity-related comorbidities.^15^ Bariatric surgery produces improvements in the CVD risk-factor profile, including metabolic syndrome, a lower risk of ischemic heart disease and mortality. Bariatric surgery also prevents new cases of diabetes compared with lifestyle treatment. A robust finding in many studies, independent of bariatric procedure, has been the improvement or remission of T2DM, before any significant WL. On the basis of several studies it is suggested that bariatric surgery serves as an alternative approach to intervention in obesity and this strategy may represent an effective treatment with concomitant reduction in T2DM, obesity-related comorbidities and reduced mortality.^51^ Bariatric surgery increases levels of total T and free T concomitant with reduction in weight, BMI and WC. Also fasting blood glucose and fasting insulin levels were significantly reduced. These findings strongly suggest that weight reduction via bariatric surgery is associated with normalization of hormonal profiles in obese men.^52^ It should be emphasized that only carefully selected patients can be subjected to bariatric surgery and patients need to be followed-up very closely and carefully. More research is needed to understand the long-term consequences of bariatric procedures in obese patients.^15^

Current strategies for the treatments for obesity include diets, exercise, behavioral lifestyle changes, pharmaco-therapeutic agents and bariatric surgery.^53, 54^ T therapy is another novel approach to treatment of obesity, as it reduces fat mass and improves lean body mass. This is of critical importance, as balance in the body composition relates to metabolic function.^55^ We believe that T therapy represents a novel pharmaco-therapeutic approach in the treatment of underlying patho-physiological conditions of obesity. We should also point out that treatment adherence is a major concern in managing chronic diseases such as obesity. We report that adherence and compliance to T therapy with TU 3-monthly injections was remarkable in previous studies. In this study, only one patient was dropped out in 8 years, due to moving to a new geographical location.

Pharmaco-therapeutics used to treat obesity must meet several important criteria, including (a) long-term WL and weight maintenance, (b) should be well tolerated and exhibit no major safety concerns, (c) patients would adhere to the therapy and remain compliant. One most noted observation is that with nonsurgical WL interventions including pharmacotherapy, most WL occurs in the first 6 months after which there is a weight plateau, or a small degree of WL or gain when followed-up for longer term. Thus, T therapy for treatment of obesity meets the aforementioned criteria. Simply, T therapy produces WL, is well -tolerated and safe, and no weight regain, and patient compliance is very high. Most importantly, this therapy improves mood, energy, vigor and overall quality of life.

It should be pointed out that labels of testosterone preparations list weight increase as a potential adverse effect of T therapy. An initial weight gain in response to T therapy may be a result of water retention, which is transient. Indeed, we measured a moderate weight gain in some of our patients after 3–6 months of T therapy, which, however, was a transient phenomenon. Only three men (<1%) had gained weight at the end of the observation time. It is, however, important that T therapy in hypogonadal men will not result in rapid WL. The US Food and Drug Administration (FDA) used to require a quick effect of weight-loss drugs, although more recently it was acknowledged that long-term effects over 1–2 years should be proven.^51^ We would like to point out that in all the reported studies to date, T therapy resulted in increase in lean body mass suggesting that T therapy should result in weight gain not WL. However, the reduction in fat mass, coupled with improved metabolic function and increased vigor and physical activity over time in response to T therapy produces the observed WL.

We should emphasize that the increases in prostate volume noted in this study were expected as hypogonadism results in decreased prostate volume and T therapy restores prostate growth to its mature size. In addition, the increases in prostate-specific antigen are also similar to that reported previously with T therapy.^56^ T therapy is always met with a number of challenges. Among these are the myths that T causes prostate cancer. Although this myth has been debunked,^56, 57, 58, 59^ the continued fear and apprehension of physicians from litigation remains a huge challenge to T therapy in men with obesity. Indeed, eight patients were diagnosed with low-grade prostate cancer in this study. This incidence rate is low compared with an untreated population of a similar age, as reported previously.^56^ It should be made clear that all patients were monitored closely, according to the guidelines of the European Association of Urology.

Other challenges including the recent hysteria regarding T therapy and cardiovascular risk was addressed in a recent comprehensive review by Morgentaler *et al.*^60^ Although such reports suffer from serious methodological flaws and poor scientific evidence, the purported information that T therapy is harmful has confounded the knowledge gained from more than seven decades of clinical experience with T therapy, and is in direct contradiction with a large body of actual patient data.^60^ Such challenges need to be overcome with concerted effort of education and scientific exchange.

### Limitations

This observational study is not without inherent limitations. We did not have a control group, due to the nature of the study. Furthermore, we do not have precise data on concomitant medication or changes in medication. We did not collect any information on lifestyle habits or the changes thereof. Finally, we did not anticipate the marked and significant WL in this study. However, the fact that WL had not been expected validates the results as patients had not gone into the study with the intention to lose weight. It should be noted that testosterone preparations are not indicated for the treatment of obesity but for hypogonadism.

## Conclusions

In this study, we report that most changes in the anthropometric parameters in response to testosterone therapy were more pronounced with increasing severity of obesity. All changes were in a clinically meaningful magnitude and sustainable for the full observation period. T therapy appears to be an effective approach to achieve sustained WL in obese hypogonadal men, thereby potentially reducing cardiometabolic risk. On the basis of the findings presented in this study, we suggest that T therapy offers safe and effective treatment strategy of obesity in men with TD and this novel approach provides a unique opportunity to manage obese hypogonadal men.

## Figures and Tables

**Table 1:** Baseline characteristics and comorbidities in 411 obese hypogonadal men according to obesity class

	*All Classes (*n=*411)*	*Class I (*n=*214)*	*Class II (*n=*150)*	*Class III (*n=*47)*
age (years (minimum; maximum))	59.46±7.05 (33;84)	58.61±8.04 (33;84)	60.35±5.73 (44;74)	60.51±5.52 (43;70)
mean follow-up (years)	6.06±1.91 (1;8)	5.91±1.92 (1;8)	6.09±1.94 (1;8)	6.66±1.67 (2;8)
				
*Anthropometry*				
weight (kg (minimum; maximum))	110.8±11.3 (86;141)	102.6±6.4 (86;129)	116.8±6.9 (95;133)	129.0±5.6 (119;141)
BMI (kg/m^2^ (minimum; maximum))	35.43±3.48 (30.1;46.51)	32.69±1.4 (30.1;34.99)	37.32±1.45 (35.01; 39.95)	41.93±1.48 (40.08;46.51)
waist circumference (cm (minimum; maximum))	110.6±8.4 (89;148)	106.8±7.4 (89;133)	113.5±7.5 (97;148)	118.5±5.6 (105;132)
				
*Glycaemic control*				
fasting glucose (mmol/L (minimum; maximum))	6.12±1.54 (3.77;12.93)	5.97±1.65 (3.77;12.93)	6.24±1.43 (3.77;12.82)	6.40±1.31 (4.94;11.99)
HbA_1c_ (% (minimum;maximum))	7.07±1.35 (4.5;11.6)	6.67±1.25 (4.6;9.7)	7.5±1.32 (4.9;11.6)	7.56±1.37 (4.5;9.4)
				
*Metabolic diseases*				
Prediabetes	103 (25.1%)	71 (33.2%)	29 (19.3%)	3 (6.4%)
Diabetes mellitus type 2	173 (42.1%)	70 (32.7%)	77 (51.3%)	26 (55.3%)
Diabetes mellitus type 1	23 (5.6%)	13 (6.1%)	8 (5.3%)	2 (4.3%)
total metabolic diseases	299 (72.7%)	154 (72.0%)	114 (76.0%)	31 (66.0%)
				
*Cardiovascular diseases*				
history of myocardial infarction	35 (8.5%)	6 (2.8%)	15 (11.5%)	11 (23.4%)
history of stroke	7 (1.7%)	2 (0.9%)	4 (3.1%)	1 (2.1%)
previous diagnosis of coronary artery disease	46 (11.2%)	23 (10.7%)	19 (14.5%)	18 (38.3%)
total cardiovascular diseases	88 (21.4%)	31 (14.5%)	38 (29%)	30 (65.0%)

**Table 2:** Changes in metabolic, prostate, and quality of life parameters at baseline and following long-term treatment with testosterone in obese hypogonadal men

	*Class I (*n=*214)*			*Class II (*n=*150)*			*Class III (*n=*47)*		
*Baseline age (years)*	*58.61±8.04 (33;84)*			*60.35±5.73 (44;74)*			*60.51±5.52 (43;70)*		
	*baseline (±s.d.)*	*8 years (±s.d.)*	*change (± s.e.)*	*baseline (±s.d.)*	*8 years (±s.d.)*	*change (±s.e.)*	*baseline (±s.d.)*	*8 years (±s.d.)*	*change (±s.e.)*
*Testosterone*									
Testosterone (nmol/L)	8.74±1.89	17.02±2.3	8.16±0.33*	9.3±1.92	16.86±2.32	7.29±0.31*	9.34±1.89	15.99±1.02	6.41±0.45*
									
*Prostate parameters*									
prostate volume (ml)	28±10.03	30.2±11.38	4.77±0.28*	33.25±8.37	36.77±9.44	3.73±0.25*	34.77±8.04	38.33±8.08	2.58±0.41*
PSA (ng/ml)	1.23±0.85	1.51±0.81	0.36±0.03*	1.63±0.85	1.9±0.74	0.35±0.03*	1.99±0.75	2.3±0.81	0.22±0.05*
IPSS	8.39±4.78	3.12±2.31	−4.33±0.23*	9.69±4.5	3.54±2.65	−6.38±0.25*	9.74±4.34	2.97±2.09	−6.93±0.33*
									
*Erectile function*									
IIEF-EF	13.87±7.23	24.83±3.53	9.13±0.39*	15.57±7.45	24.25±4.64	7.56±0.41*	17.09±7.8	25.36±3.5	6.86±0.56*
									
*Quality of life*									
AMS	53.3±10.05	19.74±4.21	−32.48± 0.7*	52.59±9.43	19.43±3.97	−33.39±0.7*	57±9.88	17.78±2.1	−38.24±1.16*
									
*Glycaemic control*									
fasting glucose (mmol/L)	5.97±1.65	4.95±0.47	−0.86±0.10*	6.24±1.43	5.08±0.46	−1.15±0.12*	6.40±1.31	5.18±0.32	−1.19±0.16*
HbA_1c_ (%)	6.67±1.25	5.37±0.4	−1.15±0.06*	7.5±1.32	5.78±0.56	−1.79±0.08*	7.56±1.37	5.68±0.44	−1.87±0.13*
									
*Lipids*									
total cholesterol (mmol/L)	7.01±1.12	4.73±0.28	−2.21±0.09*	7.60±1.04	4.78±0.25	−2.80±0.10*	7.94±1.10	4.81±0.23	−3.07±0.15*
HDL (mmol/L)	1.21±0.41	1.67±0.37	0.54±0.03*	1.45±0.50	1.99±0.48	0.57±0.03*	1.60±0.51	2.18±0.41	0.57±0.05*
LDL (mmol/L)	4.14±0.84	2.48±0.79	−1.31±0.06*	4.53±0.72	2.89±0.73	−1.46±0.07*	4.95±0.94	3.13±0.64	−1.71±0.11*
triglycerides (mmol/L)	2.98±0.68	2.04±0.23	−0.90±0.05*	3.32±0.66	2.10±0.16	−1.21±0.05*	3.72±0.68	2.15±0.12	−1.56±0.09*
total cholesterol : HDL ratio	6.45±2.44	2.96±0.59	−3.68±0.17*	6.07±2.87	2.55±0.62	−3.51±0.17*	5.66±2.88	2.29±0.47	−3.06±0.21*
									
*Erythropoiesis/Blood count*									
haemoglobin (g/dl)	14.49±0.95	15.08±0.47	0.59±2.68*	14.5±0.86	14.74±1.13	0.27±0.08^#^	14.36±0.72	15.03±0.41	0.7±0.09*
haematocrit (%)	43.32±3.49	48.14±1.78	4.38±0.33*	43.64±3.49	48.26±1.59	4.54±0.34*	43.3±2.81	48.25±1.33	4.8±0.47*
leukocytes (10^9^/L)	6.67±1.39	6.39±0.51	−0.17± 0.14	7.18±1.93	6.2±0.54	−1.01±0.17*	6.99±1.5	6.53±0.3	−0.45±0.21^§^
									
*Liver transaminases*									
AST (U/L)	29.97±10.37	18.17±5.25	−10.47±1*	36.84±14.32	17.65±5.74	−20.06±1.22*	42.28±17.68	15.26±3.58	−27.95±2.21*
ALT (U/L)	32.96±15.09	18.31±7.83	−11.99±1.39*	38.78±18.01	17.05±5.83	−22.84±1.54*	43.38±20.53	15.15±4.27	−29.44±2.77*
									
*Inflammation*									
CRP (mg/dl)	2.15±2.23	0.34±0.34	−1.81±0.14*	3.23±4.39	0.41±0.65	−3.39±0.28*	3.73±4.28	0.29±0.31	−3.99±0.42*
									
*Blood pressure*									
systolic (mm Hg)	144.3±14.59	125.91±8.37	−21.57±0.83*	157.39±15	129.15±8.13	−31.11±1.01*	161.3±14.25	132.7±8.12	−33.15±1.44*
diastolic (mm Hg)	85.2±10.38	74.18±4.69	−12.52±0.73*	93.54±12.03	74.33±5.44	−20.62±1.41*	97.06±10.79	75.97±5.43	−23.51±1.35*

Abbreviations:

ALT, alanine transaminase; AMS, Aging Males' Symptom scale; AST, aspartate aminotransferase; CRP, C-reactive protein; HDL, high-density lipoprotein; IIEF-EF, international index of erectile function, erectile function; IPSS, International Prostate Symptom Score; LDL, low-density lipoprotein; PSA, prostate-specific antigen.

*P<0.0001 vs baseline; ^#^*P*=0.0014; ^§^*P*=0.0319.

**Table 3:** Changes in weight and waist circumference from baseline to last observation (A and B); proportion of patients in categories of waist circumference at baseline and end point (C); proportion of patients in categories of BMI at last observation (D)

	*Class I (*n=*214)*		*Class II (*n=*150)*		*Class III (*n=*47)*	
	n	*%*	n	*%*	n	*%*
*A. Weight change*						
Unchanged	1	0.5	0	0	0	0
Gained	3	1.4	0	0	0	0
Any weight loss	210	98.1	150	100	47	100
Weight loss ⩾5%	200	93.5	147	98	47	100
Weight loss ⩾10%	144	67.3	134	89.3	45	95.7
Weight loss ⩾15%	85	39.7	108	72	42	89.4
Weight loss ⩾20%	35	16.4	65	43.3	28	59.6
Weight loss ⩾25%	6	2.8	19	12.7	11	23.4
Weight loss ⩾30%	0	0	3	2	0	0
						
*B. Waist circumference change*						
Unchanged	1	0.5	0	0	0	0
Gained	1	0.5	0	0	0	0
Any reduction in waist circumference	212	99.1	150	100	47	100
Reduction ⩾5 cm	192	89.7	144	96	47	100
Reduction ⩾10 cm	101	47.2	110	73.3	42	89.4
Reduction ⩾15 cm	27	12.6	51	34	15	31.9
Reduction ⩾20 cm	6	2.8	12	8	2	4.3
Reduction ⩾25 cm	1	0.5	3	2	1	2.1
						
*C. Proportion of patients in categories of waist circumference*						
Baseline waist circumference ⩾94 cm	212	99.1	150	100	47	100
Baseline waist circumference ⩾102 cm	165	77.1	148	98.7	47	100
End waist circumference ⩾94 cm	166	77.6	142	94.7	47	100
End waist circumference ⩾102 cm	34	15.9	58	38.7	37	72.3
End waist circumference <94 cm	48	22.4	8	5.3	0	0
End waist circumference <102 cm	180	84.1	92	6.1	10	21.3
						
*D. Proportion of patients in categories of BMI at last observation*						
Normal weight	9	4.2	0	0	0	0
Overweight	155	72.4	67	44.7	3	6.4
Obesity class I	49	22.9	76	50.7	35	74.5
Obesity class II	1	0.5	7	4.7	9	19.1
Obesity class III	0	0	0	0	0	0

**Table 4:** Changes of anthropometric, metabolic, prostate and quality of life parameters in obese hypogonadal men receiving testosterone treatment according to age group

*Baseline age (years)*	*Class I (*n=*214)*		*Class II (*n=*150)*		*Class III (*n=*47)*	
	*⩽65 (*n=*163)*	*>65 (*n=*51)*	*⩽65 (*n=*120)*	*>65 (*n=*30)*	*⩽65 (*n=*40)*	*>65 (*n=*7)*
*Anthropometry*	*Change±s.e.*	*Change±s.e.*	*Change±s.e.*	*Change±s.e.*	*Change±s.e.*	*Change±s.e.*
Weight (kg)	−17.5±0.5*	−16.7±1.1*	−25.7±0.6*	−24.2±1.1*	−30.4±0.7*	−31.4±2.4*
BMI (kg m^−2^)	−5.55±0.16*	−5.34±0.36*	−8.26±0.19*	−7.76±0.35*	−9.94±0.31*	−10.18±0.72*
Weight loss (%)	−16.9±0.46*	−16.16±1.01*	−21.92±0.46*	−20.58±0.94*	−23.55±0.53*	−23.97±1.6*
Waist circumference (cm)	−10.3±0.3*	−11.7±0.8*	−14.1±0.4*	−13.0±0.7*	−14.4±0.4*	−14.3±1.2*
						
*Testosterone*						
Testosterone (nmol l^−1^)	8±0.36*	8.66±0.8*	7.06±0.35*	8.09±0.68*	6.26±0.48*	7.42±1.29*
						
*Prostate parameters*						
Prostate volume (ml)	5.08±0.31*	3.41±0.66*	3.81±0.3*	3.51±0.41*	2.79±0.34*	1.05±2.26
PSA (ng ml^−1^)	0.39±0.03*	0.27±0.08^#^	0.33±0.04*	0.44±0.07*	0.29±0.04*	−0.18±0.24
IPSS	−3.89±0.25*	−6.09±0.51*	−6.35±0.3*	−6.52±0.43*	−6.75±0.35*	−8.01±0.82*
						
*Erectile function*						
IIEF-EF	8.83±0.42*	10.26±1.02*	7.71±0.48*	6.95±0.74*	7.26±0.61*	4.06±1.35**
						
*Quality of life*						
AMS	−32.58±0.78*	−32.15±1.6*	−32.85±0.79*	−35.42±1.52*	−38.62±1.18*	−36.04±4.37*
						
*Glycaemic control*						
Fasting glucose (mmol l^−1^)	−0.74±0.09*	−1.25±0.32^§^	−0.95±0.12*	−1.81±0.28*	−1.03±0.13*	−2.14±0.87^##^
HbA_1c_ (%)	−1.12±0.07*	−1.25±0.16*	−1.74±0.09*	−2.03±0.14*	−1.84±0.14*	−2.02±0.36*
						
*Lipids*						
Total cholesterol (mmol l^−1^)	−2.14±0.10*	−2.47±0.23*	−2.76±0.11*	−2.98±0.23*	−3.10±0.15*	−2.77±0.57*
HDL (mmol l^−1^)	0.57±0.03*	0.42±0.08*	0.60±0.04*	0.45±0.06"	0.55±0.05*	0.71±0.14*
LDL (mmol l^−1^)	−1.30±0.07*	−1.43±0.12*	−1.43±0.08*	−1.60±0.17*	−1.78±0.10*	−1.27±0.43^§§^
Triglycerides (mmol l^−1^)	−0.86±0.06*	−1.07±0.14*	−1.14±0.06*	−1.48±0.12*	−1.51±0.09*	−1.79±0.28*
Total cholesterol:HDL ratio	−3.77±0.19*	−3.28±0.34*	−3.59±0.19*	−3.2±0.33*	−3±0.23*	−3.44±0.59*
						
*Erythropoiesis/blood count*						
Haemoglobin (g dl^−1^)	0.49±3.39	0.66±0.22^$^	0.26±0.09^‡^	0.3±0.21	0.62±0.09*	1.18±0.31^$$^
Haematocrit (%)	4.22±0.36*	5.03±0.79*	4.74±0.36*	3.89±0.85*	4.54±0.5*	6.49±1.38*
Leukocytes (10^9^ l^−1^)	−0.29±0.16	0.21±0.32	−0.85±0.2*	−1.6±0.38*	−0.38±0.22	−0.87±0.64
						
*Liver transaminases*						
AST (U l^−1^)	−10.9±1.1*	−9.46±2.4^§^	−20.6±1.41*	−18.32±2.47*	−27.5±2.23*	−30.35±8.59^††^
ALT (U l^−1^)	−12.22±1.47*	−11.79±3.7^†^	−22.67±1.68*	−23.26±3.69*	−27.93±2.82*	−38.42±10.31^‡‡^
						
*Inflammation*						
CRP (mg dl^−1^)	−1.91±0.16*	−1.47±0.24*	−3.29±0.32*	−3.74±0.55*	−3.91±0.47*	−4.53±0.93*
						
*Blood pressure*						
Systolic (mm Hg)	−19.68±0.83*	−28.91±2.36*	−32.25±1.17*	−26.93±1.88*	−33.05±1.57*	−33.51±3.43*
Diastolic (mm Hg)	−11.6±0.77*	−15.88±1.86*	−20.34±1.78*	−21.51±1.41*	−22.84±1.51*	−27.84±2.7*

Abbreviations:

ALT, alanine transaminase; AMS, Aging Males' Symptom scale; AST, aspartate aminotransferase; BMI, body mass index; CRP, C-reactive protein; HDL, high-density lipoprotein; IIEF-EF, international index of erectile function, erectile function; IPSS, International Prostate Symptom Score; LDL, low-density lipoprotein; PSA, prostate-specific antigen.

*P<0.0001 vs baseline; ^#^*P*=0.0008; ^§^*P*=0.0001; ^$^*P*=0.0031; ^†^*P*=0.0016; ^‡^*P*=0.0037; ^**^P=0.0048; ^##^*P*=0.0185; ^§§^*P*=0.0057; ^$$^*P*=0.0005; ^††^*P*=0.0012; ^‡‡^*P*=0.0007.
